# Blowing off steam—Identifying bowel perforation after routine colonoscopy

**DOI:** 10.1002/ccr3.4515

**Published:** 2021-07-23

**Authors:** Murtaza S. Hussain, Smit S. Deliwala, Rohit Gupta, Mohammed Berrou

**Affiliations:** ^1^ Department of Internal Medicine Michigan State University at Hurley Medical Center Flint MI USA; ^2^ Department of Internal Medicine/Pediatrics Michigan State University at Hurley Medical Center Flint MI USA; ^3^ Division of Pulmonary/Critical Care Department of Internal Medicine Michigan State University at Hurley Medical Center Flint MI USA

**Keywords:** colonoscopy, endoscopy, perforation, peritonitis

## Abstract

Colonoscopy is an effective procedure for colorectal cancer (CRC) screening. Perforation is a rare yet the most severe complication, identified by landmarks—double dolphin, triangle, or double‐wall sign signifying air and intracolonic contents leaking into the peritoneal space. Prompt recognition and surgical intervention are imperative to avoid high mortality.

## Graphical abstract

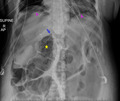

## 1 CASE HISTORY

Colonoscopy is an effective procedure for colorectal cancer (CRC) screening. Perforation is a rare yet the most severe complication, identified by landmarks—double dolphin, triangle, or double‐wall sign signifying air and intracolonic contents leaking into the peritoneal space. Prompt recognition and surgical intervention are imperative to avoid high mortality.

## 2 QUESTIONS

Which post‐colonoscopy complication is associated with a high mortality rate?

## 3 ANSWER

We present a case of bowel perforation after a routine colonoscopy

**FIGURE 1:**
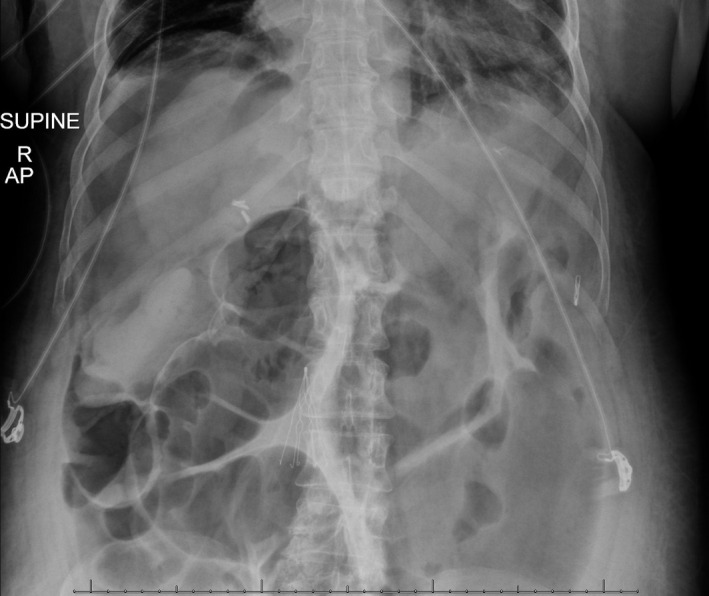
Abdominal radiograph revealing signs of abdominal perforation

**FIGURE 2:**
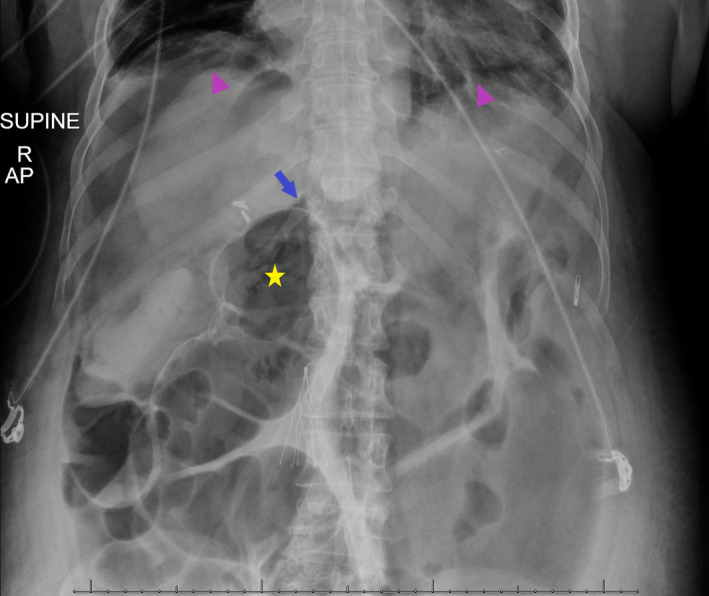
Abdominal radiograph in a supine position demonstrating classic signs of pneumoperitoneum – double dolphin sign (arrowhead), triangle sign (arrow), and double wall (Rigler) sign (star)

**FIGURE 3:**
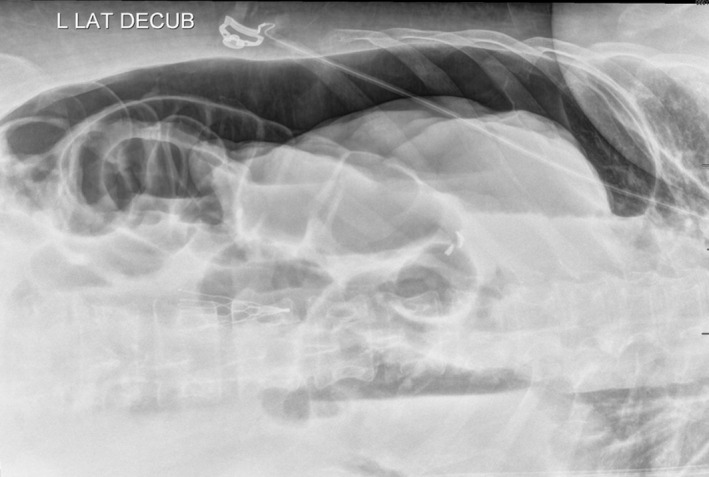
Abdominal radiograph in left lateral decubitus position

**FIGURE 4:**
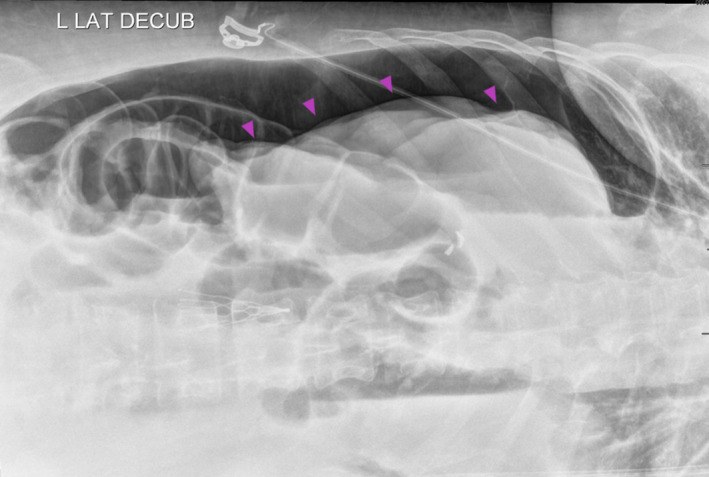
Plain abdominal radiograph in left lateral decubitus position demonstrating free air in the peritoneum (arrowhead)

A 66‐year‐old woman underwent routine colonoscopy for chronic diarrhea evaluation. Colonoscopy revealed non‐continuous large ulcers extending from the splenic flexure down to the sigmoid colon, with biopsies confirming tubular adenoma, chronic non‐specific inflammation, and ulceration. Immediately afterward, she reported severe abdominal pain and distention, with vital signs revealing hemodynamic instability. An emergent abdominal radiograph demonstrated features consistent with pneumoperitoneum on the background of viscus perforation (Figures 1 and 2).^1^ An abdominal radiograph from a different viewpoint revealed free air in the peritoneal space (Figures 3 and 4). The patient was taken to the operating room for emergent exploratory laparotomy, sigmoid colectomy with end colostomy.^2^ Intra‐procedurally, three areas of serosal tears near the proximal rectum and sigmoid colon were noted. The resected specimen confirmed colonic perforation, focal ulceration, and serosal acute inflammatory exudate. Her course eventually improved and she was eventually discharged with close follow‐up.

## CONFLICT OF INTEREST

None declared.

## AUTHOR CONTRIBUTIONS

MSH and MB involved in acquisition and review. SSD involved in conception, draft, and review. RG involved in acquisition, draft, and review.

## CONSENT STATMENT

Published with written consent of the patient.

## Data Availability

Data sharing not applicable–no new data generated, or the article describes entirely theoretical research.
